# Magnetic MgFeO@BC Derived from Rice Husk as Peroxymonosulfate Activator for Sulfamethoxazole Degradation: Performance and Reaction Mechanism

**DOI:** 10.3390/ijms252111768

**Published:** 2024-11-01

**Authors:** Tong Liu, Chen-Xuan Li, Xing Chen, Yihan Chen, Kangping Cui, Qiang Wei

**Affiliations:** 1 School of Resources and Environmental Engineering, Hefei University of Technology, Hefei 230009, China; aimee777@ustc.edu.cn (T.L.); cxli@hfut.edu.cn (C.-X.L.); xingchen@hfut.edu.cn (X.C.); yhchen@hfut.edu.cn (Y.C.); 2 Key Laboratory of Nanominerals and Pollution Control of Higher Education Institutes, Hefei University of Technology, Hefei 230009, China; 3 CAS Key Laboratory of Urban Pollutant Conversion, Department of Environmental Science and Engineering, University of Science and Technology of China, Hefei 230026, China

**Keywords:** biochar-based catalysts, sulfate radicals, peroxymonosulfate, nonradical pathway

## Abstract

Heterogeneous Mg-Fe oxide/biochar (MgFeO@BC) nanocomposites were synthesized by a co-precipitation method and used as biochar-based catalysts to activate peroxymonosulfate (PMS) for sulfamethoxazole (SMX) removal. The optimal conditions for SMX degradation were examined as follows: pH 7.0, MgFeO@BC of 0.4 g/L, PMS concentration of 0.6 mM and SMX concentration of 10.0 mg/L at 25 ℃. In the MgFeO@BC/PMS system, the removal efficiency of SMX was 99.0% in water within 40 min under optimal conditions. In the MgFeO@BC/PMS system, the removal efficiencies of tetracycline (TC), cephalexin (CEX), ciprofloxacin (CIP), 4-chloro-3-methyl phenol (CMP) and SMX within 40 min are 95.3%, 98.4%, 98.2%, 97.5% and 99.0%, respectively. The radical quenching experiments and electron spin resonance (ESR) analysis suggested that both non-radical pathway and radical pathway advanced SMX degradation. SMX was oxidized by sulfate radicals (SO_4_^•−^), hydroxyl radicals (•OH) and singlet oxygen (^1^O_2_), and SO_4_^•−^ acted as the main active species. MgFeO@BC exhibits a higher current density, and therefore, a higher electron migration rate and redox capacity. Due to the large number of available binding sites on the surface of MgFeO@BC and the low amount of ion leaching during the catalytic reaction, the system has good anti-interference ability and stability. Finally, the intermediates of SMX were detected.

## 1. Introduction

Sulfamethoxazole (SMX) is widely used as a sulfonamide antibiotic and can efficiently treat infectious diseases of humans and animals [1,2]. The pathways of SMX into the ecosystem mainly include insufficiently treated wastewater discharge, pharmaceutical industry wastewater discharge, agricultural activities and leakages from septic systems [3]. Generally, SMX is released into the aquatic environment via effluent discharge from pharmaceutical manufacturing plants. Because of the potential carcinogenicity of SMX, it has serious effects on human health [4,5,6]. Therefore, the development of pollutant degradation technology has received more attention [7].

Persulfate-based advanced oxidation processes (PS-AOPs) were verified early to be efficient for in situ chemical oxidation of contaminant due to its unique superiority such as higher oxidation potential and wider applicability under the environments [8,9,10]. Unlike H_2_O_2_, solid peroxymonosulfate (PMS) is easy to be stored, transported, and delivered [11]. The reactivity of peroxymonosulfate (PMS) is higher than peroxodisulfate (PDS) due to the structural asymmetry [12]. Peroxymonosulfate (PMS) can be activated by ultrasound, electrolysis, UV light, heating and transition metals [4,13]. Carbon-based materials have the advantages of low cost, abundant surface functionality and superior thermal stability [12]. Therefore, carbon-based materials could act as the well PMS activator and promote the removal of sulfamethoxazole (SMX).

Biochar (BC), as one of typical carbon-based materials, is derived from thermal pyrolysis of biomass feedstock, such as sludge, crop residues and animal litters [14]. BC is featured with abundant surface functional groups (e.g., C-OH, -COOH, C=O) and a developed porous structure [11,15]. In addition, persistent free radicals (PFRs) on the surface of biochar (BC) have proved to be responsible for the catalytic capacity of catalysts [11]. Jung et al. reported that MgO-modified biochar composites derived from Mg-enriched biomass show superior adsorption performance due to its increased available binding sites [16]. However, the difficulty of separating MgO-modified biochar from reaction solutions has yet to be resolved, which has limited large-scale application of MgO-modified biochar to wastewater remediation. Qin et al. reported that Fe_3_O_4_ had been used in a Fenton-like process, which exhibited a high magnetic behavior [17]. Compared with Fe_3_O_4_, Fe-Mg oxide loaded on biochar can reduce secondary pollution released by Fe [18]. Magnesium ferrite is a well-known cubic spinel ferrite nanoparticle, wherein the tetrahedral and octahedral cation sites are coordinated and occupied by divalent Mg^2+^ and trivalent Fe^3+^, respectively. Therefore, Fe-Mg oxide loaded on biochar is more stable [19].

In this work, green, efficient, non-toxic and recyclable Fe-Mg oxide co-doped biochar (MgFeO@BC) was synthetized by a co-precipitation method and used as a PMS activator for the removal of SMX. In the MgFeO@BC/PMS system, the removal efficiency of SMX was 99.0% in water within 40 min under optimal conditions. Batch experiments were performed to investigate the catalytic performance of MgFeO@BC. Moreover, the reactive oxygen species (ROS) in the MgFeO@BC/PMS system were identified by radical quenching tests and electron spin resonance (ESR) tests. The activation mechanism of PMS and degradation pathways of SMX were discussed. Finally, the practical application prospect of the MgFeO@BC/PMS system was evaluated through the interference experiment of anion and natural organic matter.

## 2. Results and Discussion

### 2.1. Characterization

**Figure 1:**
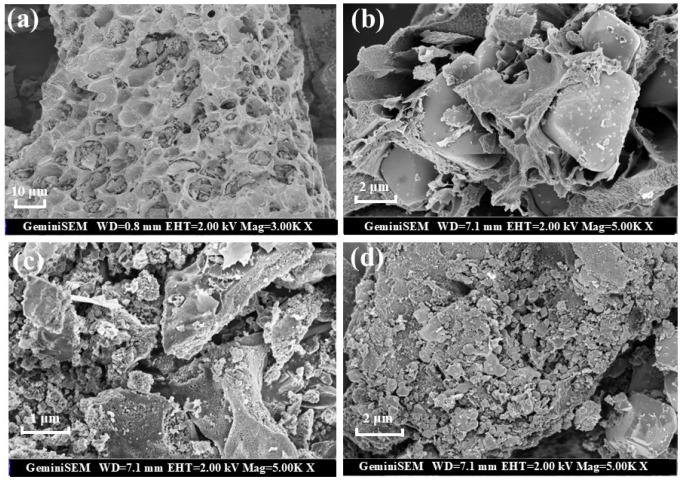
SEM images of BC (**a**), Fe_3_O_4_@BC (**b**), MgO@BC (**c**) and MgFeO@BC (**d**).

The morphology and microstructure of samples were explored using scanning electron microscopy (SEM) (Hitachi, Tokyo, Japan). The surface of primary biochar (BC) is honeycomb, which provides a sufficient platform for the loading of bimetallic oxides (Figure 1a). The loading of Fe_3_O_4_ and MgO significantly changes the morphology of biochar. There is an obvious loading of oxide particles on the surface of Fe_3_O_4_@BC and MgO@BC, but the distribution of these oxide particles on the surface of carbon-based materials is not uniform (Figure 1b,c). There are many uneven nanoparticles uniformly distributed on the surface, so the catalyst obtains more active centers and catalytic sites. The BET surface area increased from 194.86 m^2^/g to 468.91 m^2^/g after MgFeO loading, while the pore volume increased from 0.111 cm^3^/g to 0.370 cm^3^/g. MgFeO@BC presents a well-developed porous structure, which is conducive to improving the adsorption and catalytic performance of the catalyst (Figure 1d).

**Figure 2:**
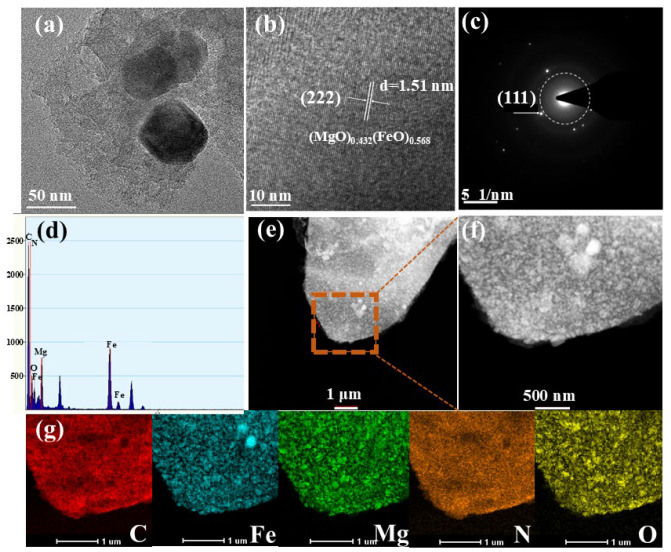
HRTEM images (**a**,**b**), SAED (**c**), EDX (**d**) and C, Fe, Mg, N, O element distribution (**e**–**g**).

The microstructure of crystal lattice of MgFeO@BC was studied in a high-resolution transmission electron microscope (HRTEM) and selected area electron diffraction (SAED) pattern. The biochar surface was successfully loaded with bimetallic oxide particles, and this transition metal oxide particles can serve as a new catalytic center for activating PMS (Figure 2a). Figure 2b proves that the carbon-based material presents a regular crystal phase structure, and the Fourier transform method is used to calculate the crystal face spacing of 1.51 nm, belonging to the (222) crystal face of (MgO)_0.432_(FeO)_0.568_. Miller concentric circles also show the crystal properties of MgFeO@BC, as shown in Figure 2c. The Miller index of (111) also proves the existence of bimetal (MgO)_0.432_(FeO)_0.568_, which is consistent with XRD analysis results. In addition, the EDX images show that there are mainly five elements on the surface of the material: C, N, O, Fe and Mg (Figure 2d). Nitrogen may be a natural element in rice husk biomass [20]. It can be seen from the distribution diagram that the distribution areas of Fe, Mg and O elements are highly overlapping and relatively uniform, which also confirms the formation and uniform loading of bimetallic oxides on the surface of biochar, as shown in Figure 2e–g.

**Figure 3:**
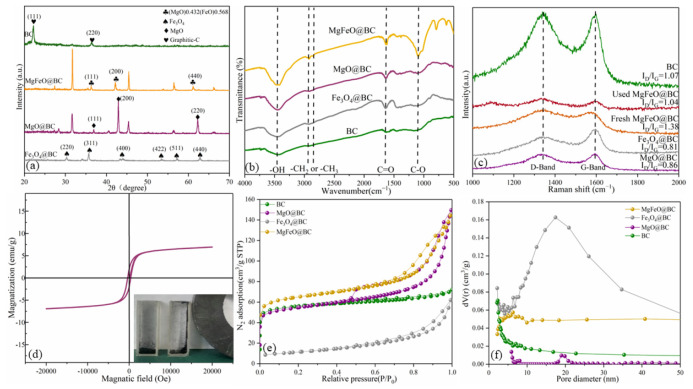
XRD patterns (**a**), FT-IR spectra (**b**), Raman spectra (**c**), room-temperature magnetization curve (**d**), nitrogen adsorption–desorption isotherms (**e**) and pore size distribution (**f**) of catalysts.

The XRD spectrum of catalysts is shown in Figure 3a. BC has distinct diffraction peaks at approximately 26.6° and 43.0°, corresponding to the graphite structure (002) and (100) crystal faces, respectively. MgO@BC shows distinct diffraction peaks at 36.8°, 42.8°, 62.1°, 74.5° and 78.4°, corresponding to the crystal plane of (111), (200), (220), (311) and (222), respectively. It was demonstrated that MgO nanoparticles had been successfully loaded onto biochar surfaces (JCPDS 89−7746). Fe_3_O_4_@BC shows distinct diffraction peaks at 30.0°, 35.4°, 43.1°, 53.4°, 56.9°, 62.5° and 74.9°, corresponding to the crystal plane of (220), (311), (400), (422), (511), (440) and (622), respectively. It was demonstrated that Fe_3_O_4_ nanoparticles had been successfully loaded onto biochar surfaces (JCPDS 89−2355). MgFeO@BC shows distinct diffraction peaks at 36.3°, 42.2°, 61.1°, 73.3° and 77.1°, corresponding to the crystal plane of (111), (200), (220), (311) and (222), respectively. By comparing with colorimetric card JCPDS 77−2368, it was confirmed that the (MgO)_0.432_(FeO)_0.568_ compound bimetallic oxide particles were loaded on the surface of biochar during the preparation of catalyst by the co-precipitation method.

The FT-IR spectrum exhibits the surface functional groups (Figure 3b). The wide peak at 3446 cm^−1^ is attributed to C-OH, the small peak at 2922 cm^−1^ is attributed to the -CH_2_ group and the absorption peak at 1636 cm^−1^ is a tensile vibration caused by the C=O/C=C group. In addition, the broad peak at 1060 cm^−1^ is attributed to the C-O group. The structural defects of MgFeO@BC are revealed by Raman spectroscopy (Figure 3c). The D band at 1340 cm^−1^ is related to the carbon matrix defect, and the G band at 1590 cm^−1^ is related to the graphite structure of the porous carbon [21,22]. Therefore, I_D_/I_G_ is considered to be a key parameter reflecting the defect degree of carbon-based materials [23,24]. I_D_/I_G_ was ranked in descending order as follows: MgFeO@BC (1.38) > BC (1.07) > MgO@BC (0.86) > Fe_3_O_4_@BC (0.81), indicating that MgFeO@BC obtained more defect sites. The XRD, FTIR and SEM/TEM images of the used catalyst are presented in Figure S1. After catalytic reaction, the crystalline structure, surface functional groups and surface morphology of MgFeO@BC did not change significantly, indicating that MgFeO@BC has high stability.

The saturation magnetization value of MgFeO@BC is 7.05 emu/g, which indicates that MgFeO@BC is relatively weak in magnetism and can effectively reduce the accumulation of iron oxides (Figure 3d). At the same time, MgFeO@BC shows a good magnetic response to magnetic fields, which helps the material to be separated from solution, ensuring the practical application potential of the system. The adsorption isotherms of MgFeO@BC, Fe_3_O_4_@BC and MgO@BC belong to type IV, while the adsorption isotherms of BC are type II. MgFeO@BC is a mesoporous structure with pore size below 50 nm (Figure 3e,f). The specific surface areas (SSAs) of MgFeO@BC (468.91 m^2^⋅g^−1^) are larger than that of Fe_3_O_4_@BC (226.39 m^2^⋅g^−1^), MgO@BC (412.55 m^2^⋅g^−1^) and BC (194.86 m^2^⋅g^−1^) (Table 1), which improves its adsorption and catalytic properties.

### 2.2. Catalytic Oxidation of SMX

**Figure 4:**
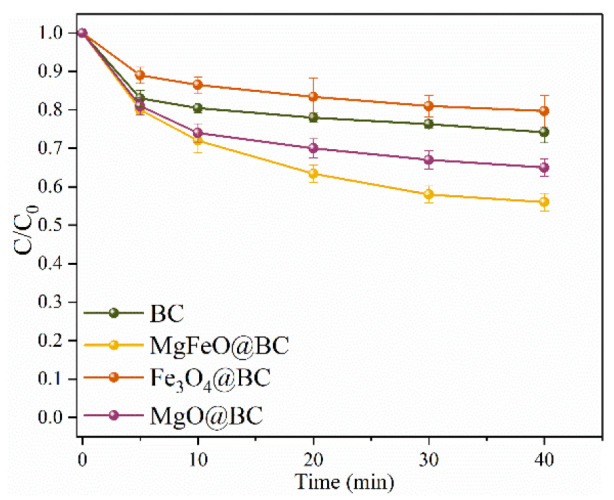
The adsorption efficiencies of SMX in various reaction systems. Reaction conditions: [SMX]_0_ = 10.0 mg/L, [catalyst]_0_ = 0.4 g/L, initial pH = 7.0 and T = 25 ℃.

The adsorption efficiencies of different catalysts for SMX are depicted in Figure 4. Under the conditions of pH = 7.0, the dosage of catalyst is 0.4 g/L, and reaction temperature is 25 ℃, the adsorption rate of MgFeO@BC, Fe_3_O_4_@BC, MgO@BC and BC for SMX is 44.0%, 20.3%, 35.0% and 25.8%, respectively, for 40 min. Combined with the results of specific surface area analysis, the higher adsorption rate of MgFeO@BC may be related to the larger specific surface area of MgFeO@BC (S_BET_ = 433.91 m^2^/g). The improvement of the adsorption properties MgFeO@BC can promote mass transfer, shorten the migration distance between PMS and SMX, and improve the degradation rate of SMX on MgFeO@BC.

**Figure 5:**
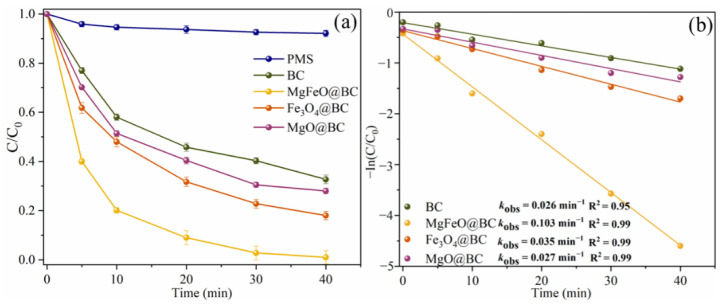
SMX degradation efficiencies (**a**), and *k*_obs_ in different systems (**b**). Reaction conditions: [SMX]_0_ = 10.0 mg/L, [catalyst]_0_ = 0.4 g/L, [PMS]_0_ = 0.6 mM, initial pH = 7.0 and T = 25 ℃.

In the absence of any catalyst, the self-decomposition rate of PMS is very low and the redox potential of PMS itself is low (1.75–1.82 V), resulting in the degradation efficiency of SMX within 40 min being only 7.9% (Figure 5a) [25]. In the BC/PMS system, the degradation efficiency of SMX was 67.3% within 40 min, indicating that biochar was not efficient enough to activate PMS to degrade SMX. The degradation efficiencies of SMX in the Fe_3_O_4_@BC/PMS system and MgO@BC/PMS system within 40 min were 82.0% and 72.0%, respectively. According to the results of the degradation experiment, MgFeO@BC can effectively promote the activation of PMS to degrade SMX, and the degradation efficiency of SMX in the MgFeO@BC/PMS system within 40 min is 99.0%. In addition, the degradation of SMX in the MgFeO@BC/PMS system conforms to pseudo-first-order kinetics, and the *k*_obs_ of 0.103 min^−1^ is 4.0 times, 2.9 times and 3.8 times that of the BC/PMS system, Fe_3_O_4_@BC/PMS system and MgO@BC/PMS system, respectively, as shown in Figure 5b. The typical pollutant degradation efficiencies in different systems are shown in Table S1.

**Figure 6:**
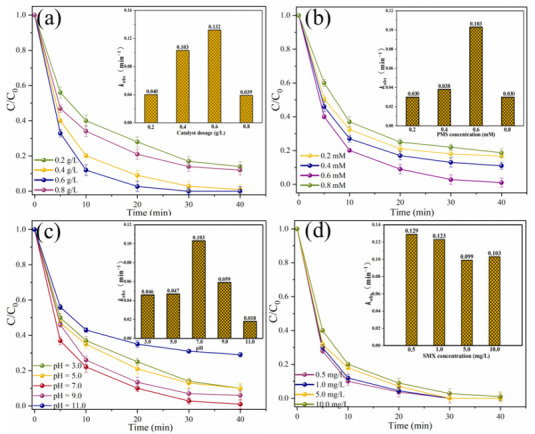
Effects of different parameters on SMX removal in MgFeO@BC/PMS system: (**a**) catalyst dosage, (**b**) PMS concentration, (**c**) solution pH, (**d**) SMX concentration. (Conditions: pH = 7.0; [SMX]_0_ = 10.0 mg/L; [catalyst] = 0.4 g/L; [PMS] = 0.6 mM; T = 25 ℃.)

The effects of different reaction parameters on the degradation efficiency of SMX were investigated (Figure 6). When the MgFeO@BC dosage is in the range of 0.2–0.6 g/L, SMX removal efficiency in MgFeO@BC/PMS system increases with the increase in MgFeO@BC dosage, from 86.0% to 100.0% (Figure 6a). The corresponding *k*_obs_ increased from 0.040 min^−1^ to 0.132 min^−1^. The increased dosage of MgFeO@BC can not only promote the interaction between pollutant molecules and MgFeO@BC, but also promote the rapid formation of reactive oxygen species (ROS), which leads to the increase in SMX degradation efficiency [26]. However, when the dosage of MgFeO@BC was increased to 0.8 g/L, the degradation efficiency of SMX decreased from 100.0% to 88.0%, and the corresponding *k*_obs_ decreased from 0.132 min^−1^ to 0.039 min^−1^. This is because the further increase in the dosage of catalyst may produce excess radicals in a short period of time, and the interaction between these radicals will lead to a decrease in the SMX removal efficiency.

When the PMS concentration increases from 0.2 mM to 0.6 mM, the degradation efficiency of SMX in the MgFeO@BC/PMS system increases significantly from 83.3% to 99.0%, and the corresponding *k*_obs_ increases from 0.030 min^−1^ to 0.103 min^−1^ (Figure 6b). Obviously, the increased concentration of PMS produces more reactive species, which leads to a higher degradation efficiency of SMX in this system. However, when the PMS concentration continued to increase to 0.8 mM, the removal efficiency of SMX decreased from 99.0% to 81.3%, and the *k*_obs_ also decreased from 0.103 min^−1^ to 0.030 min^−1^. This is because excess PMS molecules may react with radicals in the system, thereby reducing the removal efficiency of SMX [27].

**Figure 7:**
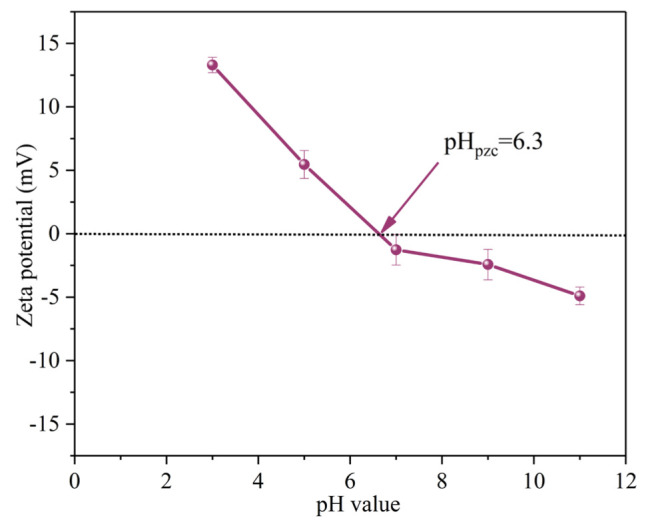
Zeta potentials of MgFeO@BC catalyst.

The initial solution pH affects the degradation efficiency of SMX by influencing the presence of pollutant molecules and PMS [28]. When the solution pH was 3.0, 5.0, 7.0, 9.0 and 11.0, the removal efficiencies of SMX within 40 min were 90.0%, 90.0%, 99.0%, 94.0% and 71.0%, respectively. The corresponding *k*_obs_ are 0.046 min^−1^, 0.047 min^−1^, 0.103 min^−1^, 0.059 min^−1^ and 0.018 min^−1^, respectively (Figure 6c). The zeta potential of MgFeO@BC is shown in Figure 7. The zero electric point (pH_zpc_) of MgFeO@BC is 6.4. When the solution is alkaline, the surface of the catalyst is negatively charged, which results in a strong electrostatic repulsion between SMX molecules and MgFeO@BC, which is not conducive to the degradation of SMX in the MgFeO@BC/PMS system. Under acidic conditions, H^+^ may consume the generated SO_4_^•−^ and •OH, which is not conducive to SMX removal [29]. A higher degradation efficiency was obtained in a wider pH range (pH = 3.0–9.0), and the most favorable degradation efficiency of SMX was achieved under neutral conditions (pH = 7.0).

The effect of the initial concentration of SMX on the removal efficiency was further explored, as shown in (Figure 6d). When the initial concentration of SMX was 0.5 mg/L, 1.0 mg/L, 5.0 mg/L and 10.0 mg/L, the removal efficiencies of SMX within 40 min were 100.0%, 100.0%, 100.0% and 99.0%, respectively. The corresponding *k*_obs_ were 0.129 min^−1^, 0.123 min^−1^, 0.099 min^−1^ and 0.103 min^−1^, respectively. The above results show that the system can degrade SMX efficiently in different concentration ranges, which also ensures that the system has practical application potential.

### 2.3. Mechanism Discussion

#### 2.3.1. Identification of Reactive Oxygen Species (ROS)

**Figure 8:**
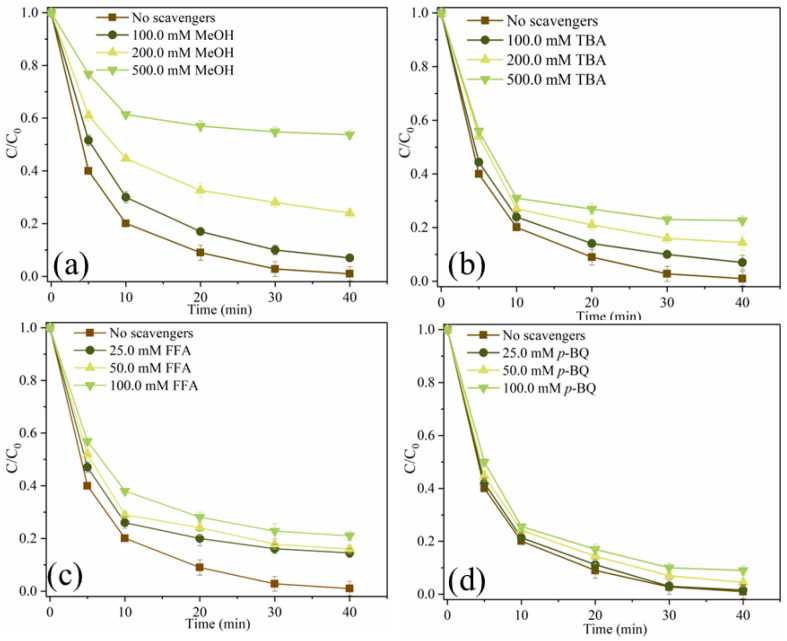
SMX degradation efficiency under different scavengers (**a**–**d**). (Conditions: [SMX]_0_ = 10.0 mg/L; [MgFeO@BC]_0_ = 0.4 g/L; [PMS]_0_ = 0.6 mM; pH = 7.0; T = 25 ℃.)

In order to investigate reactive oxygen species (ROS), which plays a dominant role in SMX degradation, the quenching experiment was carried out. Methanol (MeOH) can simultaneously remove •OH ((1.2–2.8) × 10^9^ M^−1^s^−1^) and SO_4_^•−^ ((1.6–7.7) × 10^7^ M^−1^s^−1^) from catalytic oxidation reactions, while *Tert*-butanol (TBA) can remove •OH (3.2 × 10^8^ M^−1^s^−1^) [11,30]. 1,4-benzoquinone (*p*-BQ) is often considered a quencher of O_2_^•−^ because it reacts with O_2_^•−^ at a rate of 1.0 × 10^9^ M^−1^s^−1^ [31]. In addition, furfuryl alcohol (FFA) is used to remove ^1^O_2_ from the system [25]. As shown in Figure 8a, when 100.0 mM, 200.0 mM and 500.0 mM MeOH were added to the system, the removal efficiencies of SMX were 93.0%, 76.0% and 46.3%, respectively. MeOH can significantly inhibit the degradation of SMX. As shown in Figure 8b, when 100.0 mM, 200.0 mM and 500.0 mM TBA were added to the system, the degradation efficiencies of SMX decreased from 99.0% to 93.0%, 85.6% and 77.4%, respectively. It can be seen that •OH and SO_4_^•−^ are produced in this system, and they participate in the degradation process of SMX, while SO_4_^•−^ dominates the free radical pathway. As depicted in Figure 8c, when 25.0 mM, 50.0 mM and 100.0 mM FFA were added to the system, the removal efficiencies of SMX were 85.5%, 84.0% and 79.0%, respectively, indicating that FFA also had a certain inhibitory effect on SMX degradation. As shown in Figure 8d, SMX degradation was almost unaffected when 25.0 mM, 50.0 mM and 100.0 mM *p*-BQ were added to the system, indicating that O_2_^•−^ was not produced in the system. These results indicate that both free radical pathways (•OH, SO_4_^•−^) and non-free radical pathways (^1^O_2_) are involved in the degradation of SMX in MgFeO@BC/PMS system, and SO_4_^•−^ is the main reactive oxygen species (ROS).

**Figure 9:**
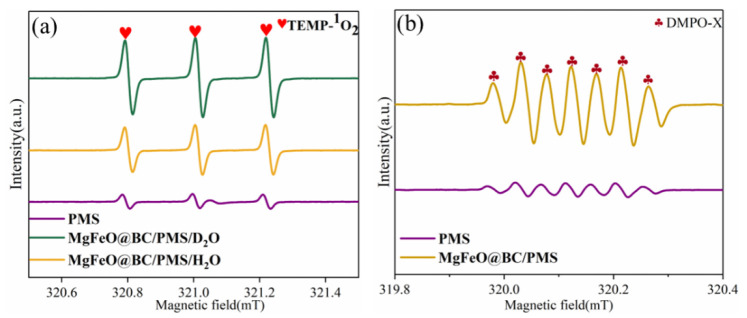
ESR signals of (**a**) TEMP-^1^O_2_, and (**b**) DMPO-X. (Conditions: [SMX]_0_ = 10.0 mg/L; [MgFeO@BC]_0_ = 0.4 g/L; [PMS]_0_ = 0.6 mM; pH = 7.0; T = 25 ℃; [TEMP] = [DMPO] = 10.0 mM.)

ESR was conducted to further verify the types of reactive oxygen species (ROS) in the MgFeO@BC/PMS system using 2,2,6,6-tetramethyl-4-piperidinol (TEMP) and 5,5-dimethyl-1-pyrroline N-oxide (DMPO) as spin-trapping agents [32,33]. When MgFeO@BC, PMS and TEMP were added into H_2_O, a typical triplet signal with the intensity ratio of 1: 1: 1 verified the existence of ^1^O_2_ (Figure 9a). The peak of TEMPO in D_2_O is higher than that in H_2_O, which implies that ^1^O_2_ rather than metastable biochar-PMS* complexes are produced in the MgFeO@BC/PMS system. When only PMS is present in the reaction solution, no obvious signal is observed, which indicates that the self-decomposition ability of PMS is weak (Figure 9b). A spectrum with seven main peaks belonging to DMPO-X was observed in the MgFeO@BC/PMS system, indicating that MgFeO@BC activated PMS to produce •OH and SO_4_^•−^.

#### 2.3.2. Reaction Mechanism

**Figure 10:**
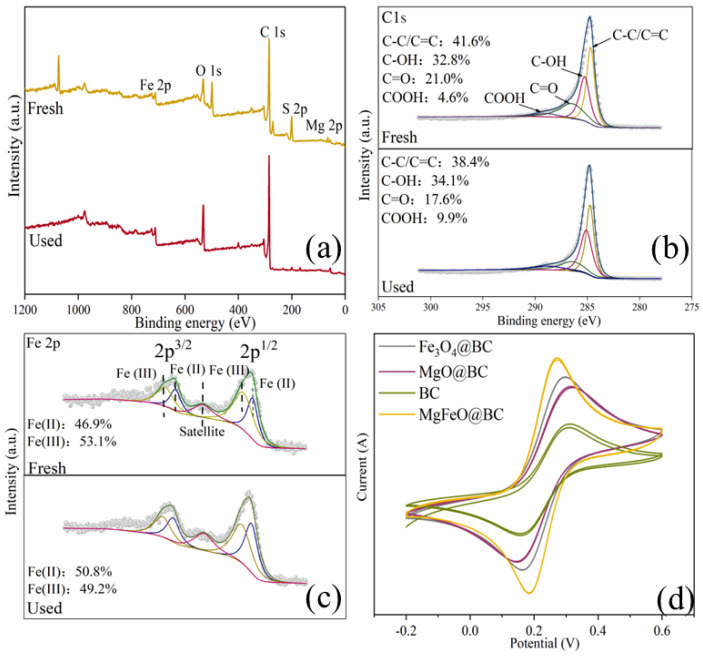
XPS spectra of full-range survey (**a**), C 1s (**b**), Fe 2p (**c**) for MgFeO@BC; CV curves of catalysts (**d**).

As depicted in Figure 10a, the full-survey-scan XPS spectrum shows five typical peaks, including C 1s, Fe 2p, O 1s, S 2p and Mg 2p, demonstrating the presence of five elements in MgFeO@BC composites. As shown in Figure 10b, four types of peaks can be fitted in the C1s spectrum: 284.8 eV (C-C/C=C), 285.6 eV (C-OH), 288.7 eV (COOH) and 287.6 eV (C=O) demonstrated the existence of multiple functional groups on the surface of the catalyst, which was consistent with the FT-IR analysis results. In the catalytic oxidation reaction, due to the large specific surface area (468.91 m^2^/g) and developed porous structure obtained by MgFeO@BC, PMS is first adsorbed on the surface of MgFeO@BC, and then enters the redox cycle between Fe (II) and Fe (III) on the catalyst surface for decomposition. The Fe 2p spectrum of MgFeO@BC is shown in Figure 10c. The three peaks at 711.3 eV, 714.1 eV and 719.2 eV belong to Fe (II), Fe (III) and Fe (satellite peak), respectively. After the reaction, Fe (II) content increased from 46.9% to 50.8%, while Fe (III) content decreased from 53.1% to 49.2%. The above results prove that part of Fe (III) transforms to Fe (II) in the catalytic reaction, Fe (III) first reacts with HSO_5_^−^ to produce Fe (II) and SO_5_^•−^ to initiate the redox reaction, and the generated Fe (II) reacts with HSO_5_^−^ to produce SO_4_^•−^ and •OH, and a complete redox cycle is formed (Equations (1)–(4)). Combined with the FT-IR analysis results, the oxygen-containing functional groups (BC-OOH, BC-OH) on MgFeO@BC can also activate PMS to produce SO_4_^•−^ (Equations (5) and (6)), and the generated reactive oxygen species (ROS) can jointly degrade SMX (Equation (7)). The cyclic voltammetry (CV) curve is shown in Figure 10d. MgFeO@BC shows a higher current density, and therefore, a higher electron migration rate and redox capacity [34].

(1) $${\mathrm{Fe}}^{3+}+{\mathrm{HSO}}_{5}^{-}\;\to {\mathrm{Fe}}^{2+}+{\mathrm{SO}}_{5}^{\cdot -}+H^{+}\;$$

(2) $${\mathrm{Fe}}^{2+}+{\mathrm{HSO}}_{5}^{-}\;\to {\mathrm{Fe}}^{3+}+{\mathrm{SO}}_{4}^{\cdot -}+{\mathrm{OH}}^{-}$$

(3) $${\mathrm{Fe}}^{2+}+{\mathrm{HSO}}_{5}^{-}\;\to {\mathrm{Fe}}^{3+}+{\mathrm{SO}}_{4}^{2-}+\cdot \mathrm{OH}\;$$

(4) $${\mathrm{SO}}_{4}^{\cdot -}+H_{2}O\;\to H^{+}+{\mathrm{SO}}_{4}^{2-}+\cdot \mathrm{OH}\;$$

(5) $$\mathrm{BC}-\mathrm{OOH}+{\mathrm{HSO}}_{5}^{-}\to {\mathrm{SO}}_{4}^{\cdot -}+\mathrm{BC}-\mathrm{OO}\cdot +H_{2}O\;$$

(6) $$\mathrm{BC}-\mathrm{OH}+{\mathrm{HSO}}_{5}^{-}\to {\mathrm{SO}}_{4}^{\cdot -}+\mathrm{BC}-O\cdot +H_{2}O\;$$

(7) $$\cdot \mathrm{OH}/{\mathrm{SO}}_{4}^{\cdot -}+\mathrm{SMX}\to \mathrm{intermediates}\;$$

### 2.4. Degradation Pathways of SMX

**Figure 11:**
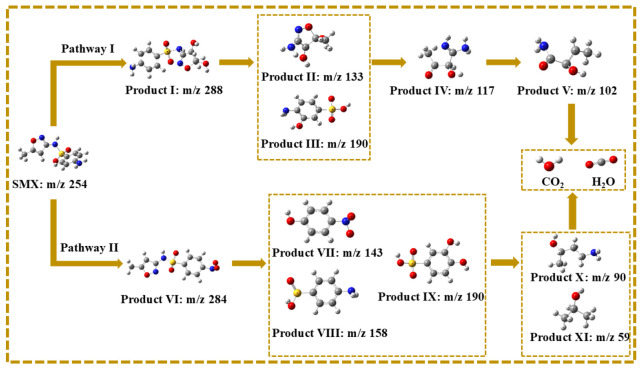
Possible pathways for degradation of SMX in MgFeO@BC/PMS system.

**Figure 12:**
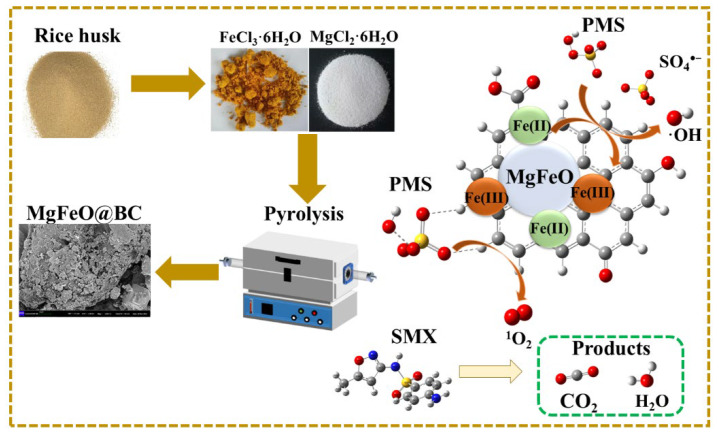
Proposed mechanism of SMX degradation in MgFeO@BC/PMS system.

The intermediates of SMX degradation were observed by ultra-performance liquid chromatography to quadrupole time-of-flight mass spectrometry (Figure S2 and Figure 11 and Table S2). The degradation pathways of SMX in the MgFeO@BC/PMS system are mainly divided into two pathways. In pathway I, the attack of SO_4_^•−^ leads to electrophilic addition of the C atom on the isoxazole ring of SMX molecule to form intermediate I (m/z = 288), followed by the break of S-N bonds to form intermediate II (m/z = 133) and intermediate III (m/z = 190). The isoxazole ring is opened to form the intermediate IV (m/z = 117), which is eventually mineralized into CO_2_ and H_2_O. In pathway II, the amino group in the benzene ring of the SMX molecule is attacked by SO_4_^•−^ to form the intermediate VI (m/z = 284). Under high redox conditions, the S-C bond of intermediate product VI is broken and hydroxylated to form intermediate product VII (m/z = 143). In addition, the S-N bond may also break and produce intermediate product VIII (m/z = 158) or intermediate product IX (m/z = 190). The benzene ring then opens to form a small molecule intermediate X (m/z = 90) and an intermediate XI (m/z = 59), which are eventually mineralized into CO_2_ and H_2_O. In summary, the degradation mechanism of SMX on the surface of MgFeO@BC is shown in Figure 12.

### 2.5. Practical Application Prospect

**Figure 13:**
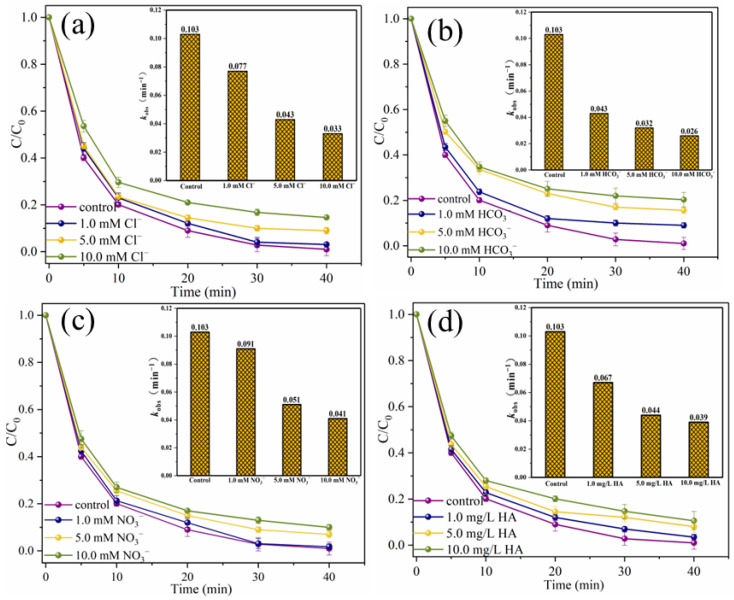
Effects of Cl^−^ (**a**), HCO_3_^−^ (**b**), NO_3_^−^ (**c**) and HA (**d**) on SMX degradation. (Conditions: [SMX]_0_ = 10.0 mg/L; [MgFeO@BC]_0_ = 0.4 g/L; [PMS]_0_ = 0.6 mM; pH = 7.0; T = 25 ℃.)

To explore the practical application prospect of the catalytic system, the interference experiments of typical anions and humic acid (HA), the degradation experiments of SMX in different water substrates, the degradation experiments of other pollutants and the ion leaching tests were carried out [35]. As shown in Figure 13a,b, when 10.0 mM Cl^−^ and HCO_3_^−^ were present in the reaction solution, SMX removal efficiencies decreased slightly from 99.0% to 85.4% and 79.7%, respectively, and the corresponding *k*_obs_ were 0.033 min^−1^ and 0.026 min^−1^, respectively. It is worth noting that when NO_3_^−^ and HA are present in the reaction system, the inhibitory effect on SMX degradation is negligible (Figure 13c,d). Specifically, when 10.0 mM NO_3_^−^ and 10.0 mg/L HA are present in the system, the removal efficiencies of SMX within 40 min are 90.0% and 89.4%, respectively. The degradation process conforms to pseudo-first-order kinetics, and the *k*_obs_ are 0.041 min^−1^ and 0.039 min^−1^, respectively. The above results showed that the presence of typical anions and HA did not significantly interfere with the degradation of SMX in this system, which might be due to the fact that there were more adsorption sites on the surface of MgFeO@BC with larger specific surface area. HA adsorbed on MgFeO@BC is insufficient to occupy too many adsorption sites and seriously hinder the degradation process of SMX in the MgFeO@BC/PMS system. At the same time, it has been reported that MgO-loaded biochar has a high binding site and can effectively remove phosphate and nitrate [17,36].

**Figure 14:**
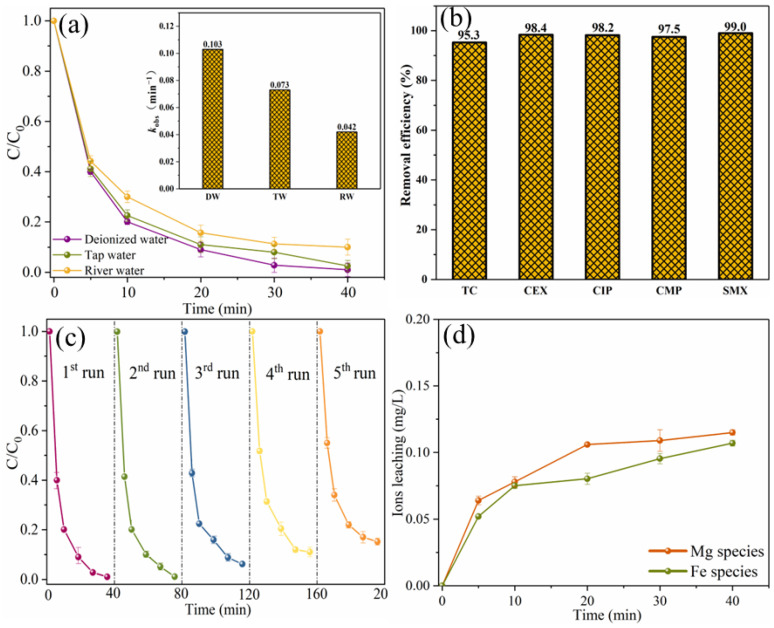
The degradation of SMX in different aqueous substrates (**a**), the removal of various pollutants in MgFeO@BC/PMS system (**b**), reusability of MgFeO@BC after 5 consecutive cycles (**c**), and the concentration of leached ions (**d**). (Conditions: [substrate]_0_ = 10.0 mg/L; [MgFeO@BC]_0_ = 0.4 g/L; [PMS]_0_ = 0.6 mM; pH = 7.0; T = 25 ℃.)

In addition, the degradation efficiency of SMX in real water was also studied, as shown in Figure 14a. The removal efficiency of SMX in tap water is slightly lower than that of ultra-pure water, and it can be almost completely removed within 40 min. The slight decrease in degradation efficiency may be due to the presence of chloride ions in the tap water. Cl^−^ could display a suppressed effect on SMX removal by consuming reactive radicals or HSO_5_^−^ [21]. In river water, the degradation efficiency of SMX decreased slightly, which may be due to the presence of a large number of inorganic anions and natural organic matter in river water [37]. However, the removal efficiency of SMX in river water within 40 min can also reach 90.3%, indicating that MgFeO@BC has strong practical application potential. In order to investigate the wide applicability of MgFeO@BC, degradation experiments of different pollutants were carried out in the same system. As shown in Figure 14b, in the MgFeO@BC/PMS system, the removal efficiencies of tetracycline (TC), cephalexin (CEX), ciprofloxacin (CIP), 4-chloro-3-methyl phenol (CMP) and SMX within 40 min are 95.3%, 98.4%, 98.2%, 97.5% and 99.0%, respectively. These results prove that the MgFeO@BC/PMS system can efficiently degrade various pollutants and has universal applicability.

To investigate the stability of MgFeO@BC, five consecutive cycle experiments were conducted, as shown in Figure 14c. The removal efficiencies of SMX were 99.0%, 97.0%, 92.0%, 89.0% and 84.8%. The main reason for reduced removal rates may be that the degradation products and their intermediates were deposited on the surface of MgFeO@BC blocking the contact between active sites and PMS [38]. Although the SMX removal efficiency decreased slightly after each cycle experiment, there was still a SMX removal efficiency higher than 80.0% after the fifth cycle experiment, indicating that MgFeO@BC has high reusability and stability in removing organic micropollutants.

Since the leaching of metal ions during the catalytic oxidation reaction may cause secondary pollution and reduce the catalytic performance, it is necessary to explore the leaching amounts of magnesium and iron ions, as shown in Figure 14d. Within 40 min, the leaching concentrations of magnesium and iron ions were 0.115 mg/L and 0.107 mg/L, respectively, which were lower than the reported leaching concentrations of MgFe_2_O_4_/BC (MMB) [18].

## 3. Materials and Methods

### 3.1. Preparation of Catalysts

MgFeO@BC was fabricated using a simple co-precipitation method. Rice husk biomass was purchased from a biomass pyrolysis power plant in China. First, the rice husks were collected and washed, dried overnight at 105 ℃, ground and sieved (0.15 mm standard test sieve). In an amount of 5.0 g, rice husks were immersed in 100.0 mL of deionized water and stirred in a constant temperature shaking bath at a speed of 200 rpm for 30 min. Then, 0.2 M MgCl_2_⋅6H_2_O and 0.4 M FeCl_3_⋅6H_2_O were added to the system, and 5.0 M NaOH was added drop by drop until the pH reached 10.0. The reaction solution was then continuously stirred at 60 ℃ for 4 h. Subsequently, the resulting composites were filtered several times and thoroughly washed to remove NaCl, and then dried at 100 ℃ for 24 h. Finally, the mixture was placed in a tube furnace and pyrolyzed in N_2_ atmosphere with a heating rate of 5 ℃/min, holding temperature of 800 ℃ and holding time of 2 h. The pyrolysis samples were cooled to room temperature under the protection of N_2_ (N_2_ flow rate: 40 mL/min), washed in deionized water several times to neutral and then dried overnight (105 ℃) to obtain the samples. The samples were named MgFeO@BC. For comparison, MgO@BC, Fe_3_O_4_@BC and BC were prepared in the same method.

### 3.2. Reaction Procedures

All tests were carried out in 50 mL beakers containing 50 mL of SMX solution (SMX concentration = 10.0 mg/L) to evaluate the catalytic performance of MgFeO@BC. An amount of 0.1 M NaOH or H_2_SO_4_ was added into the reaction solution to adjust the initial pH values before reaction. All experiments were performed in triplicate. The range of MgFeO@BC dosing was 0.2–0.8 g/L, the concentration of PMS was 0.2–0.8 mM, the pH was 3.0–11.0, SMX concentration was 0.5–10.0 mg/L and the reaction temperature was set to 25 ℃. All tests were carried out at 150 rpm, and 1.0 mL reaction solution was obtained periodically at intervals and filtered through a 0.45 μm filter. The excess reaction was terminated by adding 1 mL Na_2_S_2_O_3_ (0.2 M). The recycling experiment was performed for 5 cycles. After each cycle, MgFeO@BC was collected and dried in an oven at 80 ℃ for 12 h for further use.

## 4. Conclusions

In this study, MgFeO@BC bimetallic composite was successfully prepared by a co-precipitation method. The practical application potential of MgFeO@BC was comprehensively investigated, and the mechanism of SMX degradation in the MgFeO@BC/PMS system was revealed. (1) MgFeO@BC has a larger specific surface area (S_BET_ = 468.91 m^2^⋅g^−1^), a developed porous structure and more defective sites (I_D_/I_G_ = 1.38), and thus obtains excellent adsorption and catalytic performance, promotes the mass transfer and shortens the migration distance between the PMS and SMX. (2) The saturation magnetization value of MgFeO@BC (7.05 emu/g) is low, and MgFeO@BC maintains good magnetic recovery performance while also reducing the accumulation of iron oxides. (3) The PMS adsorbed on the surface of MgFeO@BC first enters into the redox cycle between Fe (II) and Fe (III) for decomposition, forming SO_4_^•−^, •OH and ^1^O_2_, among which SO_4_^•−^ is the most active species in the system.

## Figures and Tables

**Table 1:** BET and BJH results of different catalysts.

Samples	S_BET_ (m^2^/g)	Mean Pore Diameter (nm)	Pore Volume (cm^3^/g)	I_D_/I_G_
BC	194.86	4.33	0.111	1.07
Fe_3_O_4_@BC	226.39	4.57	0.222	0.81
MgO@BC	412.55	5.89	0.365	1.02
MgFeO@BC	468.91	6.01	0.370	2.14

## Data Availability

The data that support the findings of this study are available from the corresponding author upon reasonable request.

## Supplementary Materials

The following supporting information can be downloaded at: https://www.mdpi.com/article/10.3390/ijms252111768/s1. References [39,40,41,42] are cited in the Supplementary Materials.
