# Supplementary material for: Magnetic MgFeO@BC Derived from Rice Husk as Peroxymonosulfate Activator for Sulfamethoxazole Degradation: Performance and Reaction Mechanism
Source: Int J Mol Sci. 2024 Nov 1;25(21):11768. doi: 10.3390/ijms252111768 (PMC11546872; doi:10.3390/ijms252111768)
Supplement: Supplementary file 1 [file ijms-25-11768-s001.zip › ijms-3285603-supplementary.pdf]

Supplementary Material

Magnetic MgFeO@BC derived from rice husk as peroxymonosulfate activator for sulfamethoxazole degradation: Performance and reaction mechanism

Tong Liu[1,2,#], Chen-Xuan Li[1,2,#], Xing Chen[1,2], Yihan Chen[1,2], Kangping Cui[1,2,*], Qiang Wei[3,*]

[1] School of Resources and Environmental Engineering, Hefei University of Technology, Hefei 230009, P.R. China

[2] Key Laboratory of Nanominerals and Pollution Control of Higher Education Institutes, Hefei University of Technology, Hefei 230009, P.R. China

[3] CAS Key Laboratory of Urban Pollutant Conversion, Department of Environmental Science and Engineering, University of Science and Technology of China, Hefei 230026, P.R. China

[#]Co-first authors: These authors contributed equally to this work.

[*]Corresponding Author.

E-mail address: cuikangping@hfut.edu.cn (K.C.)

weiqiang07@ustc.edu.cn (Q.W.)

Full postal address: School of Resources and Environmental Engineering, Hefei University of Technology, No. 193 Tunxi Road, Hefei 230009, P.R. China

Department of Environmental Science and Engineering, University of Science and Technology of China, No. 96 Jinzhai Road, Hefei 230026, P.R. China

Tel: +86 0551 62901541; +86 0551 63492298

Reagents and chemicals

The chemical reagents employed in this work include sulfamethoxazole (SMX, 98.0%, Rhawn), tetracycline hydrochloride (TC, 96.0%, Rhawn), 4-chloro-3-methyl phenol (CMP, 99.0%, Rhawn), cephalexin (CEX, 98.0%, Rhawn), ciprofloxacin (CIP, 98.0%, Rhawn), sodium thiosulfate ($Na_2S_2O_3$, 99.0%, Rhawn), *L*-histidine (*L*-his, 98.0%, Rhawn), *tert*-Butanol (TBA, ≥99.5%, Rhawn), methanol (MeOH, ≥99.5%, Rhawn), and *p*-Benzoquinone (*p*-BQ, ≥99.0%, Rhawn), furfuryl alcohol (FFA, ≥98.0%, Aladdin), hydrochloric acid (HCl, 36.0−38.0%, Rhawn), sulfuric acid (H_2SO_4, 98.0%, Rhawn), sodium hydroxide (NaOH, 97.0%, Rhawn), sodium chloride (NaCl, 99.5%, Aladdin), sodium bicarbonate ($NaHCO_3$, 99.8%, Aladdin), humic acid (HA, 98.0%, Aladdin), peroxymonosulfate ($KHSO_5 \cdot 0.5KHSO_4 \cdot 0.5K_2SO_4$, Oxone, Aladdin), 5,5-Dimethyl-1-pyrroline-N-oxide (DMPO, 97.0%, Rhawn), 2,2,6,6-tetramethyl-4-piperidinol (TEMP, ≥98.0%, Aladdin). All chemicals were employed without further purification and deionized water (18.2 MΩ/cm) was used throughout the experiments.

Characterization and analysis methods

Morphologies of MgFeO@BC were observed by the scanning electron microscope (SEM, Hitachi SU8020) and high-resolution transmission electron microscope (HR-TEM, JEOL JEM-2100). The crystalline structure of MgFeO@BC was detected by the X-ray diffractometer (PANalytical, Netherlands). ATR-FTIR spectra were obtained by a FT-IR spectrometer (Thermo, Nicolet 6700) in the 4,000-500 cm^{-1} range. The chemical states of MgFeO@BC were analyzed by X-ray photoelectron spectroscopy (XPS, Thermo, ESCALAB250Xi). The SSAs and pore size distributions were analyzed with the Brunauer-Emmett-Teller (BET) method (Autosorb-IQ3, Quantachrome, USA). Raman spectrometer (HORIBA JOBIN YVON, LabRAM HR Evolution) was used to acquire Raman spectra.

SMX concentration was determined by high-performance liquid chromatography (HPLC, Shimadzu, LC-20AT). The mobile phase was SMX of acetonitrile/deionized water (0.1% formic acid) (70%/30%, v/v) at a flow-rate of 1.0 $mL \cdot min^{-1}$. The wavelength and column temperature were set to 255 nm and 30°C, respectively. Total organic carbon (TOC) was measured using a Shimadzu TOC-vcph analyzer (Multi N/C 3000). The zeta potential of MgFeO@BC was determined with a Zetasizer NanoBrook Omni (Brookhaven). Radical studies were performed with a JES-FA200 (JEOL) spectrometer. The intermediates were detected with ultra-performance liquid chromatography to quadrupole time-of-flight mass spectrometry (UPLC-TOF/MS, ACQUITY UPLC LCT Premier XE, America).

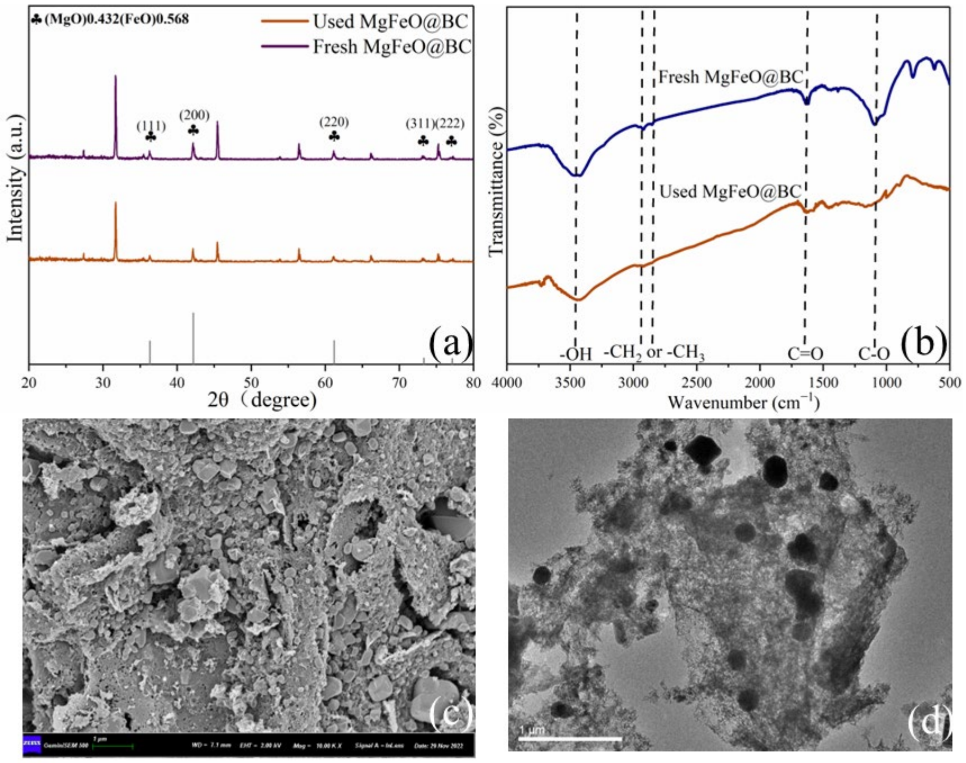

Fig. S1. XRD pattern (a), FT-IR spectrum (b), SEM image (c), HRTEM image (d) of the used MgFeO@BC.

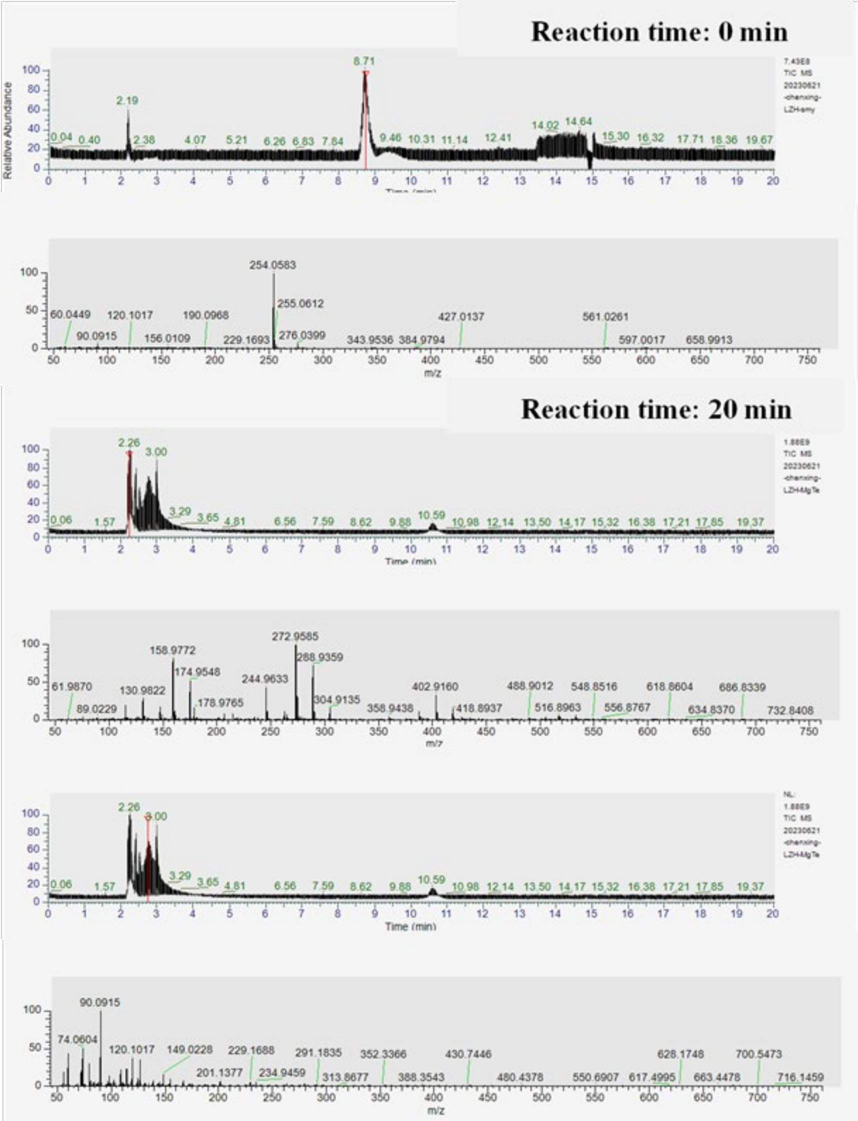

Fig. S2. Mass spectra of intermediate products.

Table S1. Studies of the typical pollutants degradation with PS activated by biochar-based catalysts.

Pollutants	Catalyst	Oxidant source	Performance
25.0 mg/L	15 mg/L Co_3O_4@NPC	0.2 mM PMS	Complete removal of sulfamethoxazole (SMX) was achieved within 5 min (Chen et al., 2022).
20.0 mg/L	2.0 g/L nanoFe_3O_4-biochar	5.0 mM PMS	Bisphenol A (BPA) removal efficiency gained 100% in 90 min at pH 3.0 with PMS 5 mM, Fe_3O_4-BC load 2.0 g/L and BPA 20 mg/L (Cui et al., 2021).
20.0 mg/L	1.0 g/L F2BC3	1.6 g/L PMS	The results showed that more than 97.0% of BPA was removed with 1.0 g/L F_2BC_3 and 1.6 g/L PMS from the artificial solution containing 20 mg/L BPA within 150 min at different pH (3, 6, and 10) (Gao et al., 2022).
2.0 mg/L	50 mg/L 0.5Fe@LSBC800	0.1 mM PMS	The optimized 0.5Fe@LSBC800 showed the optimum catalytic activity and almost complete carbamazepine (CBZ) was removed in the 0.5Fe@LSBC800/PMS system within 30 min (Gou et al., 2023).
0.2 mM	100 mg/L Cobalt-impregnated spent coffee ground biochar (Co-SCG)	0.6 mM PMS	Tetracycline (TC) was almost completely degraded in 25 min with a rate constant of 17.78×10^{-2} min^{-1} under the following optimal condition (Nguyen et al., 2019).

Table S2. Intermediates of SMX degradation detected by ultra-performance liquid chromatography to quadrupole time-of-flight mass spectrometry.

Structural formula	Molecular formula	m/z
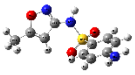	$C_{10}H_{11}N_3O_3S$	254
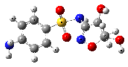	$C_{10}H_{12}N_3O_5S$	288
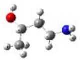	$C_4H_{11}NO$	90
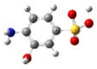	$C_6H_7NO_4S$	190
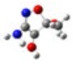	$C_4H_7N_2O_3$	133

References

Chen, Y., Bai, X., Ji, Y., Shen, T., 2022. Reduced graphene oxide-supported hollow Co3O4@N-doped porous carbon as peroxymonosulfate activator for sulfamethoxazole degradation. Chemical Engineering Journal 430.

Cui, X., Zhang, S.-S., Geng, Y., Zhen, J., Zhan, J., Cao, C., Ni, S.-Q., 2021. Synergistic catalysis by Fe3O4-biochar/peroxymonosulfate system for the removal of bisphenol a. Separation and Purification Technology 276.

Gao, Y., Chen, Y., Song, T., Su, R., Luo, J., 2022. Activated peroxymonosulfate with ferric chloride-modified biochar to degrade bisphenol A: Characteristics, influencing factors, reaction mechanism and reuse performance. Separation and Purification Technology 300.

Gou, G., Huang, Y., Wang, Y., Liu, C., Li, N., Lai, B., Xiang, X., Li, J., 2023. Peroxymonosulfate activation through magnetic Fe3C/Fe doped biochar from natural loofah sponges for carbamazepine degradation. Separation and Purification Technology 306.

Nguyen, V.T., Nguyen, T.B., Chen, C.W., Hung, C.M., Huang, C.P., Dong, C.D., 2019. Cobalt-impregnated biochar (Co-SCG) for heterogeneous activation of peroxymonosulfate for removal of tetracycline in water. Bioresour Technol 292, 121954.
